# DEK associates with tumor stage and outcome in HPV16 positive oropharyngeal squamous cell carcinoma

**DOI:** 10.18632/oncotarget.15582

**Published:** 2017-02-21

**Authors:** Eric A. Smith, Bhavna Kumar, Kakajan Komurov, Stephen M. Smith, Nicole V. Brown, Songzhu Zhao, Pawan Kumar, Theodoros N. Teknos, Susanne I. Wells

**Affiliations:** ^1^ Cancer and Blood Diseases Institute, Cincinnati Children's Hospital Medical Center, Cincinnati, OH, 45229, USA; ^2^ Department of Otolaryngology–Head and Neck Surgery, The Ohio State University, Columbus, OH, 43210, USA; ^3^ The Ohio State University, Comprehensive Cancer Center, Columbus, OH 43210, USA; ^4^ Department of Pathology, The Ohio State University, Columbus, OH, 43210, USA; ^5^ Center for Biostatistics, The Ohio State University, Columbus, OH, 43210, USA

**Keywords:** DEK, oropharyngeal squamous cell carcinoma, human papillomavirus, IL6, p16

## Abstract

Oropharyngeal squamous cell carcinomas (OPSCC) are common, have poor outcomes, and comprise two biologically and clinically distinct diseases. While OPSCC that arise from human papillomavirus infections (HPV+) have better overall survival than their HPV- counterparts, the incidence of HPV+ OPSCC is increasing dramatically, affecting younger individuals which are often left with life-long co-morbidities from aggressive treatment. To identify patients which do poorly versus those who might benefit from milder regimens, risk-stratifying biomarkers are now needed within this population. One potential marker is the DEK oncoprotein, whose transcriptional upregulation in most malignancies is associated with chemotherapy resistance, advanced tumor stage, and worse outcomes. Herein, a retrospective case study was performed on DEK protein expression in therapy-naïve surgical resections from 194 OPSCC patients. We found that DEK was associated with advanced tumor stage, increased hazard of death, and interleukin IL6 expression in HPV16+ disease. Surprisingly, DEK levels in HPV16- OPSCC were not associated with advanced tumor stage or increased hazard of death. Overall, these findings mark HPV16- OPSCC as an exceptional malignancy were DEK expression does not correlate with outcome, and support the potential prognostic utility of DEK to identify aggressive HPV16+ disease.

## INTRODUCTION

Each year over 500,000 new cases of head and neck cancers (HNCs) are reported worldwide [1], 40,000 of which occur in the United States [2]. There are two biologically distinct subtypes of these malignancies: human papillomavirus positive (HPV+) and negative (HPV-) [3]. HPV- disease predominates in older HNC patients with a history of long-term alcohol and tobacco use and is declining in prevalence alongside a decrease in smoking habits [4, 5]. This translates to a decrease in HNC at anatomical sites that predominately harbor HPV- tumors. However, the incidence for HPV+ HNC, particularly in oropharyngeal squamous cell carcinomas (OPSCC), has increased by 58% in the past two decades [6–9]. Even with the generous assumption that all eligible people receive immunization against HPV, this upward trend is still expected to continue until the vaccinated generation comes of age in 30–40 years [9]. Meanwhile, developing new innovations to characterize and treat OPSCC, the form of HNC most likely to harbor HPV, will be a necessity.

In general, patients with HPV+ malignancies have a decreased risk of disease progression, respond better to therapy, and have overall better survival compared to HPV- malignances [10–12]. However, HPV+ disease occurs in younger patients and the morbidities associated with aggressive surgical and chemoradiation therapy can reduce their quality of life dramatically [13, 14]. Because of their more favorable prognosis, there are efforts to de-escalate treatment regimens to avoid these morbidities, but it is unclear which patients will respond optimally [12]. Additionally, a sub-population of patients with advanced T4 or N3 stage HPV+ OPSCCs do poorly with standard treatment and have a 5-year survival rate of 54% [15]. In order to identify high-risk HPV+ OPSCCs, further stratification of their clinical and biological characteristics is needed, together with the identification of new biomarkers indicative of more aggressive disease.

HPV is the most prevalent sexually transmitted virus, well-known to induce cervical cancer [16], and the high-risk HPV16 serotype is the cause of almost 90% of HPV+ OPSCC [17, 18]. While just over 50% of all OPSCC are HPV+ [8], the mechanism for how HPV+ tumors respond more favorably to treatment [10, 11] is still incompletely understood [19]. However, inhibition of p53 [20] and retinoblastoma (RB) pocket proteins [21] by the HPV E6 and E7 oncoproteins, respectively, are likely at play as the p53 and RB tumor suppressors often remain intact during malignant transformation [22]. Because of the sustained expression of E6 and E7, HPV+ OPSCC require fewer somatic mutations to develop tumors [23–25], may not have developed the complement of mutations necessary for chemoradiation therapy resistance, and can still respond to treatment by activating p53, RB family members, and other interacting tumor suppressors [26]. In both HPV+ and HPV- OPSCC, early stage non-invasive tumors can be resected with good outcomes (> 80% survival) [27]. Unfortunately, the majority of cases are diagnosed at advanced, locally invasive stages where chemoradiotherapy response and overall survival decrease [28]. Generally, patients with HPV+ disease respond better than their HPV– counterparts even at these advanced stages; however, HPV+ tumors with large primary (T4) and/or large tumor extensions into lymph node (N3) still carry a relatively poor survival prognosis compared to early stage disease [29].

One method of determining the HPV status of OPSCC is by immunohistochemical staining for the presence of p16 (CDKN2A). In OPSCC there is a high degree of correlation between p16 and HPV infection [30], but there are false positives which can be ruled out with high-sensitivity *in-situ* hybridization (ISH) or polymerase chain reaction for viral nucleic acids [30–33]. The p16 gene product is a well-known tumor suppressor capable of inducing senescence in both OPSCC and primary keratinocytes, the normal epidermal cell type from which OPSCC tumors originate [34–36]. In these systems, p16 drives senescence by stabilizing and preventing the inactivation of the RB pocket proteins RB1, p107, and p130. Should these factors be inactivated, such as by E7 expression during HPV infection, then p16 expression can rise dramatically in the absence of senescence induction and even acquires oncogenic functions [37–39]. In HPV– OPSCC disease, p16 is commonly mutated, deleted, or silenced through promoter methylation [35, 40]. In general, p16 is considered a good surrogate marker for HPV infection. However, there is a subset of HPV– OPSCC that are p16 positive, whose clinical and biological characteristics are not well studied [30, 32, 33].

Inactivation of the retinoblastoma proteins by HPV also drives the expression of other oncogenic factors through E2F-mediated transcription [22, 41]. One important oncogene in many tumors upregulated in this manner is DEK [42]. This oncogene was originally described as a DEK-CAN (NUP214) fusion protein in t(6:9) acute myeloid leukemia [43] and is a highly conserved DNA binding protein in vertebrates with no known paralogs. In normal cells, this protein has functions in DNA replication [44], mRNA splicing [45], chromatin remodeling [44, 46, 47], and DNA repair [48, 49]. In normal keratinocytes, DEK overexpression has been shown to promote hyperplasia and proliferation [50, 51], inhibit differentiation [51], induce mitotic defects and chromosome abnormalities [52], block apoptosis [53], and drive transformation in cells expressing HPV E6 and E7 [53]. While the molecular mechanisms whereby this oncogene promotes these phenotypes remain surprisingly unclear, it is thought that DEK functions through chromatin binding/modification [54]. Due to the importance of DEK for these varied oncogenic phenotypes, the near-ubiquity of high DEK expression in most cancers [55], the similarity of phenotypes observed across tumor types [55], and the ability of the protein to be secreted by cells [56], DEK is currently being evaluated as a biomarker for bladder carcinoma and other malignancies [57].

Compared to normal tissue, DEK expression is upregulated in most surveyed tumor types, including breast [58, 59], hepatocellular carcinoma [60], colorectal cancer [61], and, recently, OPSCC [50]. Our previous study found that DEK protein was highly expressed in all of a small subset of 21 HPV+ and HPV– OPSCC samples that were analyzed [50]. To validate these initial results and establish a more refined relationship between DEK expression and HPV status, we surveyed a large population of OPSCC patients using an established set of primary OPSCC tissue microarrays (TMAs). We first examined the association of DEK with HPV based on p16 (CDKN2A) status and HPV16 genome *in-situ* hybridization (ISH). Following this analysis, the association of DEK with IL6 expression was also tested. IL6 is a pro-inflammatory interleukin that is strongly associated with poor overall OPSCC patient survival [62] and increased risk of metastasis [63]. Even though IL6 was found to be transcriptionally downregulated following loss of DEK in HNC tissue culture models [64], a link between DEK and IL6 expression in patient OPSCC tumors has yet to be elucidated.

In this study, we found that elevated DEK expression associates with IL6 expression, higher stage tumors, and worse prognosis in HPV16+ OPSCC. These findings support further work into developing DEK as a biomarker for HPV16+ disease and are in agreement with the DEK biomarker literature. Surprisingly, however, our data do not support similar conclusions for HPV16 negative OPSCC as there was no association between DEK expression and survival or tumor stage. This potentially marks HPV16- OPSCC as one of few solid tumors where DEK is not useful as a biomarker, and may indicate distinct biological activities for this protein in the development and progression of HPV16+ versus HPV16- disease.

## RESULTS

For this retrospective case study, 194 patients were enrolled, and the cohort represented the expected demographics of OPSCC disease (Table 1). Approximately 57% of this cohort had HPV16+ disease, as determined by genomic HPV *in-situ* hybridization (ISH), which was expected from a previous epidemiological study [8]. In line with the prognostic focus of this work, the collected tumor specimens were treatment naïve and obtained from initial surgery with curative intent. Overall the cohort experienced a 56.0% survival rate with 34.9% and 71.8% in the HPV16- and HPV16+ groups, respectively (Supplementary Table 1).

**Table 1:** Patient and tumor sample characteristics

Patient Characteristics	Mean	SD
Age (years)	57.6	9.8
	*n*	%
**Gender**		
Male	158	81.4
Female	36	18.6
**Race**		
African American/Black	9	4.6
White	185	95.4
**Marital Status**		
Single/Divorced/Widowed	80	45.2
Married	97	54.8
**HPV16 Status**		
Negative	83	43.0
Positive	110	57.0
**Smoking Status: Pack Years**		
10 pack years or less	47	25.3
More than 10 pack years	139	74.7
**Node Stage**		
N0/N1	70	36.1
N2/N3	124	63.9
**Tumor Stage**		
T1/T2	122	62.9
T3/T4	72	37.1
**AJCC Stage**		
I	4	2.1
II	10	5.1
IIII	47	24.2
IV	133	68.6
**Recurrence Status**		
No Recurrence	131	72.0
Recurrence	51	28.0
**Recurrence Type**		
Distant	19	37.3
Locoregional	32	62.7
**Extranodal Extension**		
No	110	58.2
Yes	79	41.8
**Perineural Invasion**		
No	143	74.1
Yes	50	25.9

### DEK is most highly expressed in HPV16+/p16+ OPSCC tumors

**Figure 1:**
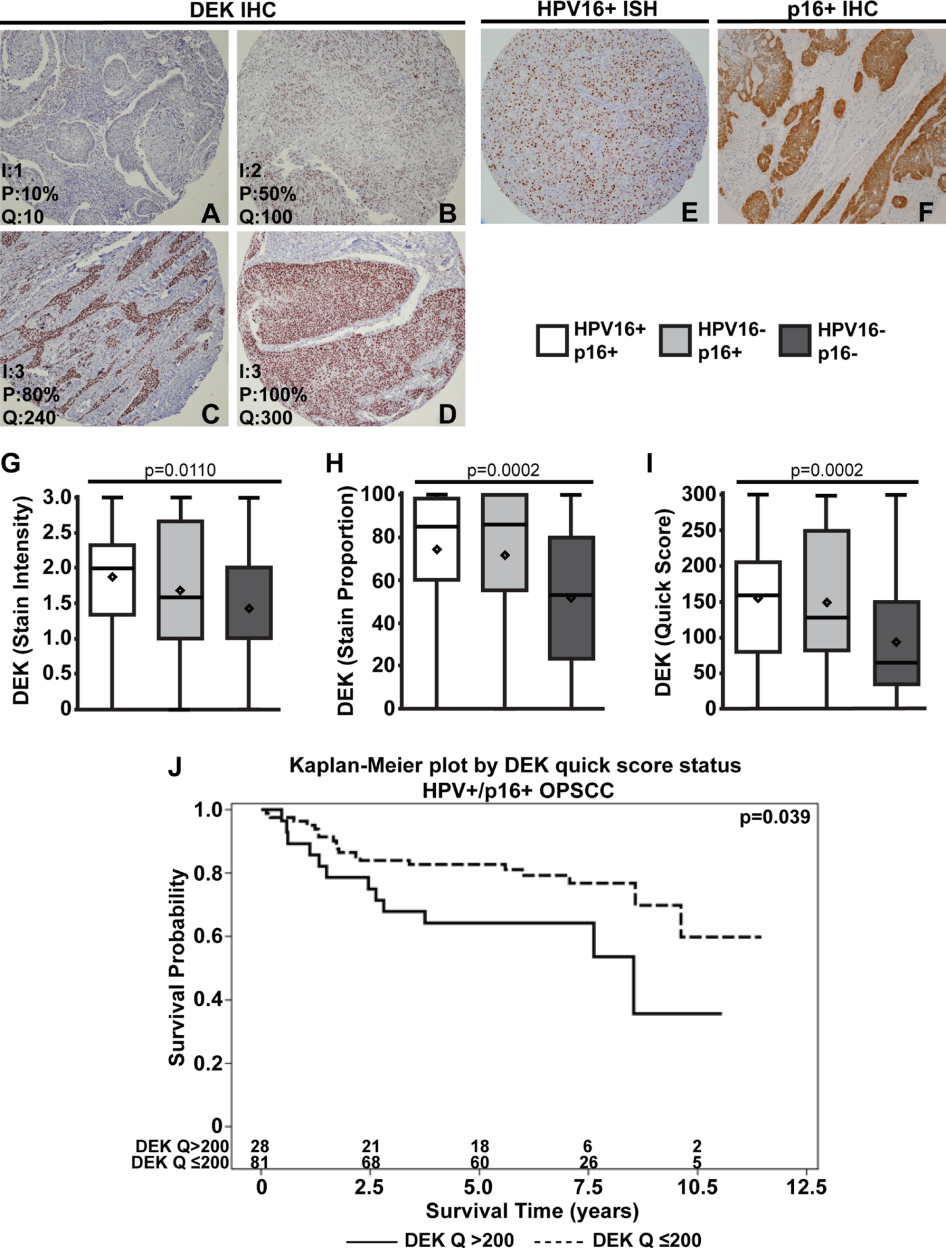
DEK is most highly expressed in HPV16+/p16+ OPSCC Tumors were scored based on DEK stain intensity (0: none, 1: low, 2: moderate, 3: high), and the proportion of tumor cells stained for DEK (0–100%). Representative images of DEK staining and quantification are as follows: low DEK staining (**A**), moderate staining (**B**), high DEK staining (**C**), and high DEK staining with complete tumor coverage (**D**) (100x magnification). Scores for intensity (I), proportion of tumor cells stained (P), and the quick score (Q, Q = IxP) are shown in the bottom left of each image. Representative images of positive stains for HPV16 ISH (**E**) and p16 IHC (**F**) are shown. After quantification, tumors were separated based on HPV16/p16 status and analyzed for differences in DEK stain intensity (**G**), proportion of cells stained (**H**), and quick score (**I**). DEK quick score dichotomized at > 200 vs. ≤ 200 is a predictor of survival in HPV16+/p16+ OPSCC (hazard ratio (HR) = 2.1, 95% CI = (1.02, 4.3), *p* = 0.039). The number at risk for each group (i.e., the number remaining for each group at a given time point) is given at the bottom of the graph. (**J**).

DEK protein expression levels and HPV16/p16 status were determined using tissue microarrays (TMAs). For each patient sample, three non-adjacent tumor tissue cores and one normal tissue core were used, and DEK staining was consistent across the three tumors cores in each case. Figure 1A–1D shows representative DEK staining patterns and quantification of stain intensity, proportion of tumor cells stained, and the calculated quick score for each image. All quantified samples were grouped based on HPV16 and p16 status; representative positive p16 stains are shown in Figure 1E–1F. There were significant differences in DEK staining across the HPV16+/p16+ (*n* = 109), HPV16-/p16+ (*n* = 36), and HPV16–/p16– (*n* = 46) OPSCC subtypes (HPV16+/p16- not shown because *n* = 1). HPV16+/p16+ OPSCC had significantly higher average DEK stain intensity (Figure 1G), stain proportion (Figure 1H), and quick score (Figure 1I) compared to double-negative HPV16–/p16– subjects (*p* < 0.01 in all cases after Bonferroni correction), while the HPV16-/p16+ group only had higher stain proportion (*p* = 0.01) and quick score (*p* = 0.03) relative to HPV16–/p16– subjects. HPV16+/p16+ and HPV16-/p16+ groups were not statistically significantly different for any of the three DEK measures after Bonferroni correction.

### DEK expression in tumors predicts advanced disease and poorer survival in HPV16+, but not in HPV16- OPSCC

**Figure 2:**
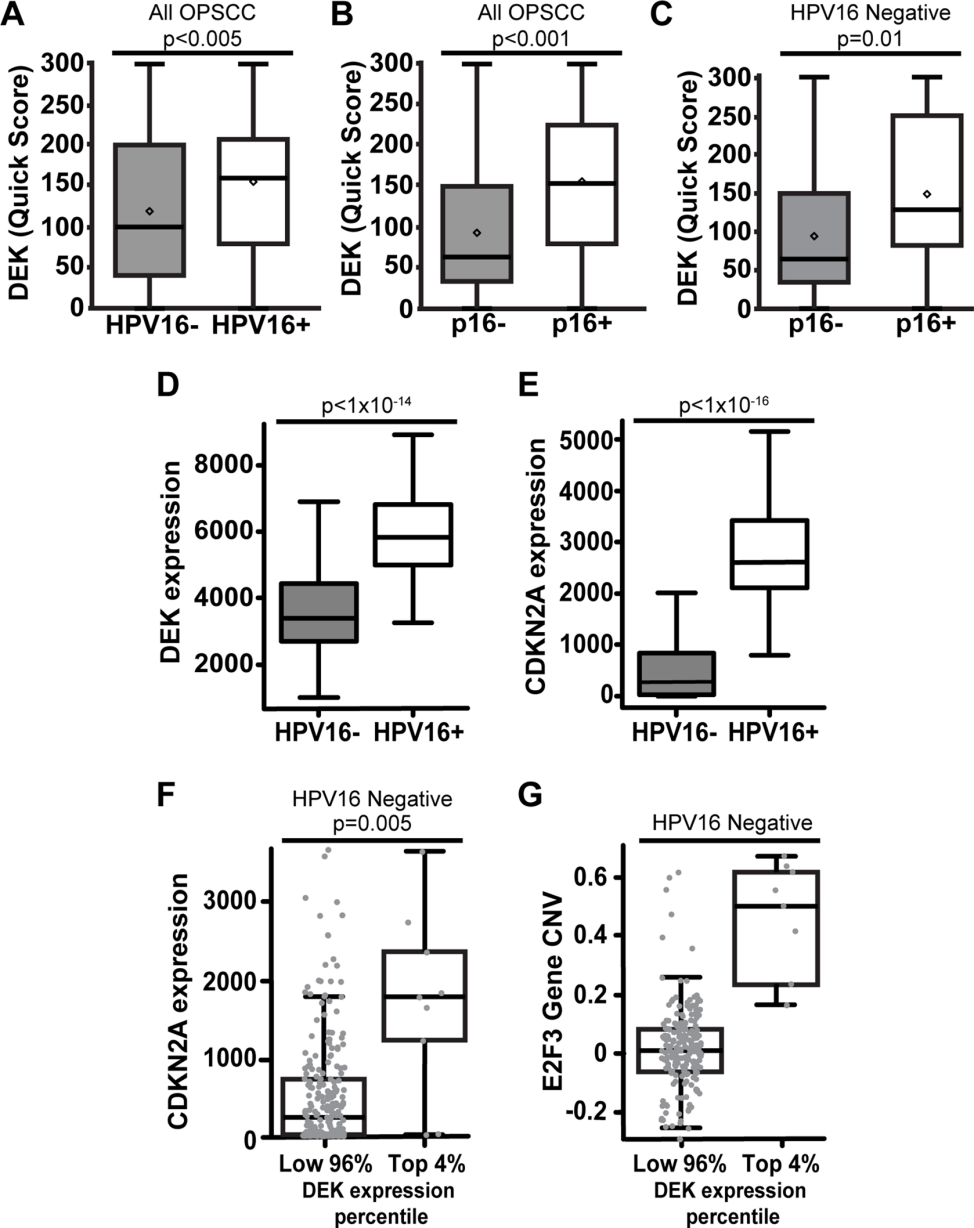
DEK expression correlates with p16+ status in both HPV+ and HPV– OPSCC Comparing all 194 OPSCC tumors, the proportion and intensity of DEK expression was significantly higher in HPV16+ (**A**) and p16+ (**B**) tumors. Quartiles and statistical significance of DEK staining intensity and tissue proportion for (A) and (B) are depicted in Supplementary Table 2. DEK expression correlated with p16 status in HPV16- tumors (**C**). D-G are an analysis of published Cancer Genome Atlas Network RNA-Seq data for HNCs [22]. DEK mRNA expression was significantly elevated in HPV+ disease (**D**). While p16 (CDKN2A) message was significantly elevated in HPV16+ disease as expected, there was substantial variability in p16 expression in HPV- tumors (**E**). The highest 4% of HPV- DEK expressing tumors (9 out of 242) were significantly increased for p16 expression (**F**). DEK was highly expressed in these tumors in correlation with amplification of DEK and the nearby E2F3 locus on 6p22.3 (**G**).

While our previous report indicated consistently high DEK staining in 21 OPSCC tumors, 17 of these were AJCC stage IV disease [50]. This current work sought to use a larger sample size, with well-represented stage I-IV disease to further assess the utility of DEK as a biomarker in OPSCC (Table 1). In this cohort, a wider range of DEK staining was noted compared to our previous study [50], indicating that DEK expression was more dynamic than initially predicted (Figure 1, Supplementary Table 1), and some of the increased variability may be due to the increased representation of early stage OPSCCs (Table 1). Tumors bearing high DEK staining and a larger percentage of DEK positive cells, as indicated by the quick score (*p* = 0.039), had an increase in the hazard of death for patients with HPV16+/p16+ malignancies (Table 2). To better clinically understand the association, Kaplan-Meier curves based on categorizing patients into two groups using the 75th percentile of the DEK quick score (DEK Q > 200 vs. DEK Q ≤ 200) are given (Figure 1J, hazard ratio (HR) = 2.1, 95% CI = (1.02, 4.3), *p <* 0.039). When the malignancies where separated based on HPV16 status alone, patients with HPV16+ tumors were much more likely to bear higher DEK quick scores than their negative counterparts (Figure 2A, Supplementary Table 2, *p* < 0.005) and maintained the increased hazard of death (Supplementary Table 3, *p < 0.039*). These results suggest DEK is a marker of worse prognosis within the subset of HPV16+ OPSCC. This hypothesis is supported by the finding that a high burden of DEK positive cells in HPV16+/p16+ specimens correlates with advanced tumor stage (Table 3, Supplementary Table 4, *p* = 0.02). In stark contrast, HPV16- OPSCCs did not show a similar correlation between high DEK expression and tumor stage (Table 3) or hazard of death (Table 2, Supplementary Table 3). DEK instead predicted a lesser degree of perineural invasion, the scientific basis of which is unknown. Importantly, the survival data mark HPV16- OPSCC, both p16+ and p16-, as a minority of solid tumors where high DEK expression does not correlate with disease stage or outcome.

**Table 2:** DEK expression is associated with an increased hazard of death in HPV16+/p16+ but not in HPV16- disease (survival univariate models)

Predictor	Hazard Ratio	95% CI		*p*-value	*N*
**HPV+/p16+**					
DEK Stain Intensity	1.518	0.964	2.392	0.0718	109
DEK Stain Proportion	1.013	0.998	1.028	0.0859	109
DEK Quick Score	1.004	1.000	1.008	**0.0388**	109
**HPV–/p16+**					
DEK Stain Intensity	0.918	0.589	1.430	0.7043	36
DEK Stain Proportion	1.002	0.990	1.015	0.7217	36
DEK Quick Score	0.999	0.995	1.003	0.6319	36
**HPV–/p16–**					
DEK Stain Intensity	0.650	0.421	1.006	0.0531	46
DEK Stain Proportion	0.993	0.982	1.004	0.2063	46
DEK Quick Score	0.997	0.993	1.002	0.2015	46

**Table 3:** High DEK expression is associated with higher tumor stage in HPV+/p16+ OPSCC, and reduced perineural invasion in HPV- OPSCC

HPV16+/p16+, DEK (Stain Proportion)							
**Tumor Stage**	**N**	**Minimum**	**25th Pctl**	**Median**	**75th Pctl**	**Maximum**	***p*-value**
T1/T2	76	0.00	53.33	75.00	98.33	100.00	**0.0227**
T3/T4	33	23.33	80.00	93.33	100.00	100.00	
**Perineural Invasion**	**N**	**Minimum**	**25th Pctl**	**Median**	**75th Pctl**	**Maximum**	***p*-value**
No	90	0.00	60.00	80.00	98.33	100.00	0.2786
Yes	19	11.67	83.33	90.00	100.00	100.00	
**HPV16–/p16+, DEK (Stain Proportion)**							
**Tumor Stage**	**N**	**Minimum**	**25th Pctl**	**Median**	**75th Pctl**	**Maximum**	***p*-value**
T1/T2	24	0.00	41.67	81.67	98.33	100.00	0.2399
T3/T4	12	5.00	65.00	92.50	100.00	100.00	
**Perineural Invasion**	**N**	**Minimum**	**25th Pctl**	**Median**	**75th Pctl**	**Maximum**	***p*-value**
No	26	0.00	63.33	89.17	100.00	100.00	0.1189
Yes	10	0.00	43.33	78.33	90.00	100.00	
**HPV16–/p16–, DEK (Stain Proportion)**							
**Tumor Stage**	**N**	**Minimum**	**25th Pctl**	**Median**	**75th Pctl**	**Maximum**	***p*-value**
T1/T2	19	0.00	23.33	53.33	76.67	98.33	0.5842
T3/T4	27	0.00	20.00	53.33	83.33	100.00	
**Perineural Invasion**	**N**	**Minimum**	**25th Pctl**	**Median**	**75th Pctl**	**Maximum**	***p*-value**
No	25	0.00	50.00	66.67	86.67	100.00	**0.0144**
Yes	20	0.00	11.67	33.33	51.67	100.00	

Statistics for other clinical characteristics listed in Supplementary Table 4.

### DEK expression is associated with p16+ status in both HPV16+ and HPV16- disease

While DEK expression did not correlate with survival in either p16+ or p16- OPSCC patients (Supplementary Tables 1, 5), DEK expression by quick score was significantly increased in p16+ tumors (*p* < 0.001, Figure 2B, Supplementary Table 2). Considering that HPV E7 disrupts and inactivates the entire RB pocket protein family, a correlation between the expression of DEK and p16 was expected as both are upregulated upon pocket protein loss of function [37, 38]. This correlation between p16 and DEK quick score was conserved in HPV16-/p16+ OPSCCs (Figure 2C). Since the percentage of HPV16-/p16+ tumors in this study (24.8% of p16+ tumors) was similar to what has been reported previously (25.7%) [32, 33], we sought to validate the relationship between p16 and DEK using published HPV– HNC Cancer Genome Atlas data [23]. HPV status was rigorously validated through a combination of whole genome, whole exome, and RNA sequencing for viral sequences, as well as ISH for HPV16, 18, 33, 35, 39, 45, 51, 52, 56, 58, and 66 serotypes. DEK and HPV status correlated with p16 (CDKN2A) expression (Figure 2D–2E), and the majority of p16+ tumors were also HPV16+, as expected (Figure 2E). However, a sizable subgroup of HPV16- tumors was identified that expressed moderate to high levels of p16 (Figure 2E–2F), with the top 4% of DEK-expressing HPV– HNCs harboring significant p16 mRNA over-expression (Figure 2F). To provide a potential mechanism for how some HPV– OPSCCs may induce p16 overexpression, we re-analyzed the TCGA data, shown in Figure 2E–2F, and found that chromosome region 6p22.3 was amplified in tumors where DEK was most highly expressed (Figure 2G). This amplified region encodes both the DEK and E2F3 genes; the latter has been implemented in driving expression of p16 [65].

### DEK correlates with IL6 expression in HPV16+ OPSCC

**Figure 3:**
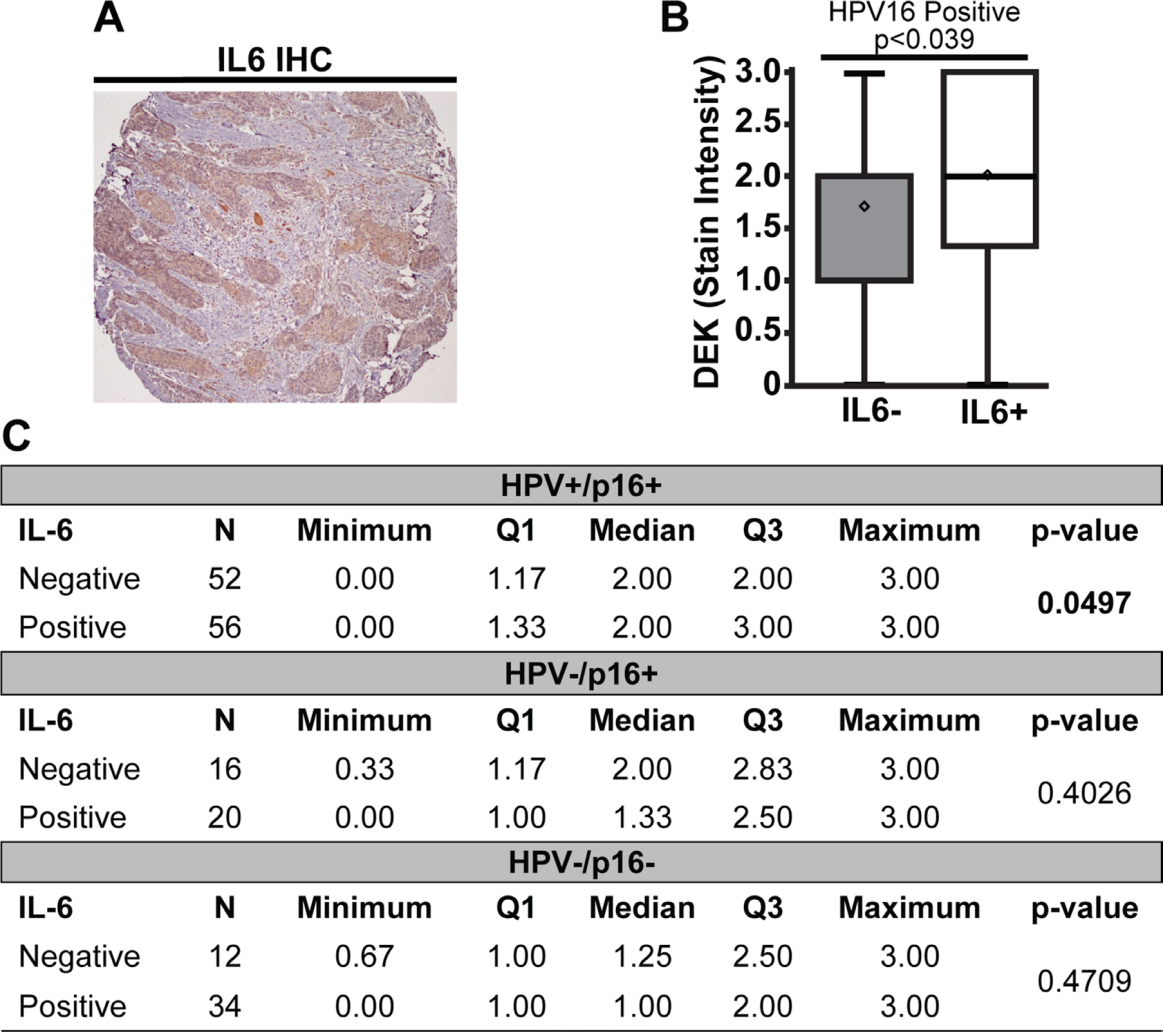
High DEK expression was associated with IL6 expression in HPV16+ tumors A representative IL6+ staining section is shown (**A**). HPV16+ tumors that were also IL6+ stained darker for DEK than tumors not expressing IL6 (**B**). There was no association between DEK and IL6 status in HPV16-/p16+ or HPV16–/p16– tumors (**C**).

In addition to promoting tumor growth and invasion, DEK has potential pro-inflammatory properties. These include autoantigen properties when secreted or present in body fluids [56, 66] and control of robust expression of inflammatory pathway members such as IRAK1 [64]. In the latter report, RNA-sequencing of HPV+ and HPV– HNC cells identified shared decreased IL6 expression in response to DEK knockdown. To determine whether DEK expression correlated with IL6 status in OPSCC, we quantified IL6 expression in the TMAs (Figure 3A). HPV16+ and HPV+/p16+ OPSCC tumors showed a correlation between high DEK stain intensity and positive IL6 status (Figure 3B–3C, *p* < 0.04 and *p* = 0.05 respectively). While this relationship was inverse in total HPV16- OPSCC (Supplementary Figure 1), this study lacked the power to confirm this association in either the HPV–/p16+ or HPV–/p16- subgroup as no significance was observed in DEK stain intensity, stain proportion, or quick score (Figure 3C, data not shown). A role for HPV16 in independently regulating both IL6 and DEK expression in HPV16+ disease cannot be ruled out by this study, and the relationship between DEK and IL6 in HPV16- OPSCC is subtle.

## DISCUSSION

Most clinical studies of DEK across human tumors have correlated high expression of the oncogene with advanced tumor stages and poor outcomes [59, 61, 67–69]. We found that this relationship holds true in HPV16+ OPSCC (Figure 1J, Table 2 and Supplementary Table 3). Considering that HPV16 accounts for approximately 90% of HPV+ OPSCCs [17, 18], these data indicate that DEK has potential as a prognostic biomarker for the vast majority of these tumors. However, this study was a medium-sized retrospective cohort designed to determine the potential for DEK as a biomarker in different OPSCC groups. To rigorously ascertain the clinical significance and prognostic value of DEK in HPV16+ disease, a large multi-institutional prospective study is now warranted.

Importantly, our data do not support DEK as a biomarker in OPSCC identified as HPV16-, HPV16-/p16+, or HPV16–/p16– (Table 2 and Supplementary Table 3). This is a surprising finding given the extensive literature on DEK expression in other HPV– solid tumor types, and suggests that HPV16- OPSCC is one of few wherein DEK does not have prognostic utility. Our data thus strongly suggests that DEK is not prognostic in all subgroups of a given malignancy, and must therefore be carefully examined as an appropriate biomarker in each case. Interestingly, DEK was significantly associated with a decreased risk of perineural invasion in the double negative group. The scientific basis of this latter finding is unknown, and should warrant further investigation as high DEK expression is usually associated with invasion and migration phenotypes [70]. With regard to the HPV16-/p16+ group, the combination of limited sample number (*n* = 36) and heterogeneity of HPV+ and HPV– tumors likely precluded any significant clinical findings. To address these issues and more deeply study HPV16-/p16+ OPSCC, a significantly larger patient population would be required.

DEK mutations cannot explain the observed disparities between HPV16+ versus HPV16- disease. Such mutations are rare in all tumors, and found in only 0.4% of OPSCC. Specifically, we analyzed the TCGA provisional head and neck cancer study, and only 2 out of 530 tumors harbored mutations within the DEK gene. The first was an E20V substitution, and the second was a nonsense frameshift at amino acid 11. No DEK mutations were identified in the 279 samples previously published by TCGA [23].

Alongside survival analysis, this report determined that the average DEK expression was highest in HPV16+ OPSCC by TMA (Figure 1G–1I) and in published TCGA data [23] (Figure 2D). This is likely due to early increases in DEK expression. DEK is upregulated by E2F transcriptional activator family members, which in turn are activated by HPV E7 disruption of RB family members [41, 42]. The additional increase in DEK protein levels which correlated with advanced stage HPV16+ tumors (Table 3) is most likely a result of HPV E7 gene amplification and increases in E7 transcriptional activation and mRNA stability. These are all common occurrences in HPV+ malignancies [71, 72]. Apart from HPV16+ tumors, the next highest DEK-expressing group was the HPV16-/p16+ cohort. This group is partially comprised of tumors bearing other high-risk HPV serotypes which would over-express DEK in the same manner as HPV16+ OPSCCs. True HPV–/p16+ tumors likely comprise the remainder of this group, and we have found that a significant fraction of HPV– tumors express p16 in the TCGA cohort (Figure 3E–3F). Other reports have also described this HPV–/p16+ population [30, 32, 33], but their clinical and biological characteristics are the least studied of OPSCC subtypes. We found that p16+ status was significantly correlated with the highest DEK-expressing HPV– tumors (Figure 2F). Biologically, this may be a consequence of chromosome 6p22.3 amplification, a common occurrence in multiple tumor types [73], but one that has not been reported in HNC until now. This region contains both the DEK and E2F3 genes, and was amplified in top DEK-expressing HPV– malignancies (Figure 2G). Currently, it is thought that one mechanism of p16 upregulation may occur through activator E2F genes, including E2F3, although the exact mechanism for this relationship is unknown [65]. It is thus possible that the amplification of E2F3 drives p16 and DEK expression.

As the field moves towards incorporating personalized medicine for HNC care, identifying and characterizing distinct subtypes of HPV+ and HPV– OPSCC will become paramount. This study contributes to this endeavor by identifying DEK as a potential prognostic biomarker for further study in HPV16+ OPSCC, and does not support its use for HPV16- disease. Prior to this current study, we and others have argued for the development of therapeutic DEK inhibiting molecules to take advantage of broad solid tumor dependency on high DEK expression for survival [53, 68, 74–76]. While DEK targeting strategies will undoubtedly be useful in the treatment of most solid tumor types, our study also suggests for the first time that use of these agents will need to be carefully tailored in the care of OPSCC patients.

## MATERIALS AND METHODS

### Study population

This retrospective case study was approved by the Ohio State University Institutional Review Board and a waiver of HIPAA authorization was obtained. All patient OPSCC specimens were requested from samples obtained by The Ohio State University James Cancer Hospital and Solove Research Institute from 2002 to 2009, and were treatment naïve at the time of collection. During the enrollment period, all patients were given the option of primary (C)RT or primary surgery with follow-up (C)RT as necessary. The majority of patients opted for initial surgery, and this is the population that was included in this study. All samples were chemo- and radiotherapy naïve, and post-surgical standard of care treatment was followed as necessary. There was no bias in selecting patient samples based on size or stage. The following patient attributes were accessed: age, race, gender, marital status, smoking status, tumor size, nodal status, AJCC stage, presence of local metastasis, presence of perineural invasion, survival time and tumor recurrence. In this report, survival time was defined as the time from the patient's primary surgical resection of OPSCC to death. The date of the last living observation was censored. Recurrence is defined as any occurrence of a new suspicious head and neck mass that is confirmed by radiology or pathology as squamous cell carcinoma within five years of surgical resection.

### Tissue microarray (TMA), immunohistochemistry (IHC), and HPV in-situ hybridization

The Ohio State University Department of Pathology Histology Core Laboratory generated master TMA blocks from selected archived paraffin-embedded tissue. Briefly, the distribution of tumor and normal tissue was determined via hematoxlin and eosin staining by a pathologist, and the TMA master blocks were created from 0.6-mm punch cores of 3 representative tumor tissues and 1 normal tissue for comparison in each specimen. TMA slides were stained for IL6 and p16, by IHC, and for HPV16, by *in-situ* hybridization (GenPoint, Dako), following previously published methods [62]. IHC staining for p16 was performed using the CINtec mtm antibody (E6H4 clone). DEK IHC staining followed standard xylene deparaffination and rehydration in decreasing ethanol concentrations. Antigens were retrieved using a Biocare Medical LLC (Concord, CA, USA) decloaking chamber with Dako antigen unmasking buffer for 20 minutes at 120°C. After returning to room temperature, slides were incubated for 10 minutes at ambient conditions with Dako dual endogenous enzyme block. This was followed with blocking in PBS/serum solution corresponding to species of the secondary antibody. Following this, BD-Pharmingen mouse anti-DEK primary antibody was applied to the TMA and incubated for 1 hour at 37°C. After washing, a room temperature 30 minute incubation was performed using biotinylated donkey anti-mouse secondary antibody (Vectastain Elite Kit). After washing, slides were incubated with an avidin-biotin complex for 30 minutes (Vector Laboratories, Burlingame, CA, USA) prior to the addition of 3,3′-diaminobenzidine (Sigma). The reaction was quenched in water, counterstained with Mayer's hematoxylin, and coverslipped with Permount.

### IHC scoring

A treatment-blinded pathologist interpreted the slides and scored for stain intensity (0: none, 1: low, 2: moderate, 3: high), and stain proportion (0–100%). A descriptive quick score (0–300) was acquired by multiplying these two dimensions. To be considered positive for p16, strong and diffuse nuclear and cytoplasmic staining in ≥ 50% of the tumor cells was required.

### Statistical methods

Descriptive statistics were used to summarize the study population, including means for the continuous variables and frequencies for the categorical variables. Cox proportional hazards models were used to assess univariate associations of DEK expression and the risk of death for the overall study population, and also stratified by HPV status, p16 status, and a combination of HPV and p16 status. Unadjusted hazard ratios and confidence intervals (CI) are reported. Mann-Whitney tests were used to assess associations between biomarkers/demographic/clinical characteristics and DEK expression (quantitative). Analyses were conducted in SAS, version 9.3 (SAS Institute, Cary, North Carolina).

## SUPPLEMENTARY MATERIALS FIGURES AND TABLES




