# Supplementary material for: DEK associates with tumor stage and outcome in HPV16 positive oropharyngeal squamous cell carcinoma
Source: Oncotarget. 2017 Feb 21;8(14):23414–26. doi: 10.18632/oncotarget.15582 (PMC5410314; doi:10.18632/oncotarget.15582)
Supplement: Supplementary file 1 [file oncotarget-08-23414-s001.pdf]

DEK associates with tumor stage and outcome in HPV16 positive oropharyngeal squamous cell carcinoma

Supplementary Materials

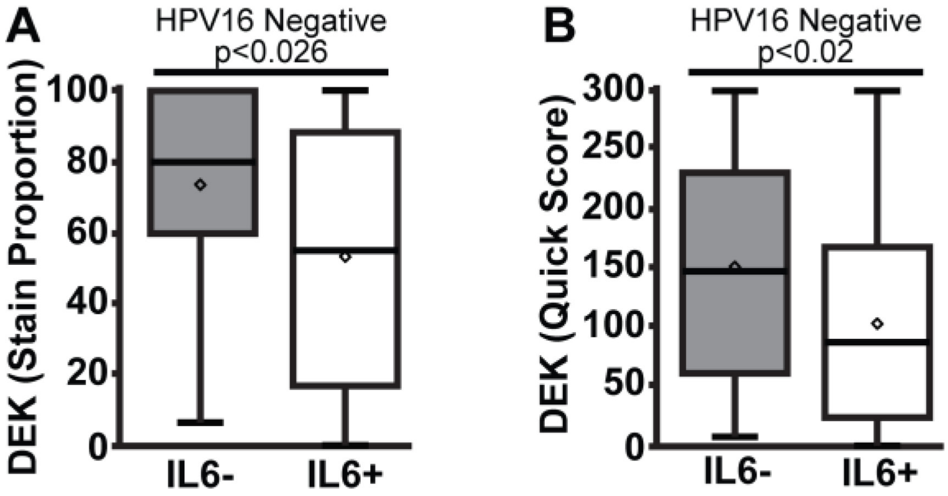

Supplementary Figure 1: High DEK expression is associated with IL6- status in HPV16- disease. The proportion of cells staining for DEK (**A**) and the overall DEK staining (**B**) was significantly lower in IL6+ tumors.

Supplementary Table 1: DEK survival summaries based on individual HPV and p16 status

	HPV–		HPV+	
	Survival		Survival	
**Biomarker**	**Alive** ***n* = 29**	**Dead** ***n* = 54**	**Alive** ***n* = 79**	**Dead** ***n* = 31**
DEK Stain Intensity				
Median (IQR)	2 (1–2.7)	1.3 (1–2)	2 (1–2.3)	2 (1.5–3)
Mean (SD)	1.8 (1.0)	1.4 (0.9)	1.8 (0.8)	2.1 (0.8)
Min – Max	0–3	0–3	0–3	0–3
DEK Stain Proportion				
Median (IQR)	75 (38.3–95)	66.7 (30–90)	80 (53.3–98.3)	90 (73.3–100)
Mean (SD)	64.1 (33.7)	58.5 (35.8)	71.4 (29.9)	80.9 (23.5)
Min – Max	0–100	0–100	0–100	0–100
DEK Quick Score				
Median (IQR)	120 (43.3–230)	96.7 (40–183.3)	140 (73.3–200)	183.3 (113.3–253.3)
Mean (SD)	137.2 (106.6)	109.4 (89.2)	145.2 (88.8)	181.0 (84.9)
Min – Max	0–300	0–300	0–300	0–300

	p16–		p16+	
	Survival		Survival	
**Biomarker**	**Alive** ***n* = 14**	**Dead** ***n* = 33**	**Alive** ***n* = 94**	**Dead** ***n* = 52**
DEK Stain Intensity				
Median (IQR)	1.3 (1–2.5)	1 (1–2)	2 (1–2.3)	2 (1.2–2.7)
Mean (SD)	1.7 (0.8)	1.3 (0.8)	1.8 (0.9)	1.9 (0.8)
Min – Max	1–3	0–3	0–3	0–3
DEK Stain Proportion				
Median (IQR)	58.3 (36.7–80)	50 (18.3–80)	81.7 (58.3–100)	88.3 (73.3–100)
Mean (SD)	58.7 (28.8)	48.1 (34.1)	71.3 (31.3)	78.5 (27.5)
Min – Max	16.7–100	0–100	0–100	0–100
DEK Quick Score				
Median (IQR)	64.2 (38.3–150)	56.7 (18.3–120)	140 (73.3–200)	166.7 (100–246.7)
Mean (SD)	111 (94)	84.7 (79.5)	148.9 (94)	167.8 (88.3)
Min – Max	23.3–300	0–266.7	0–300	0–300

Supplementary Table 2: DEK staining intensity, staining proportion, and quick score correlation with HPV and p16 status in all tumors

DEK (Stain Intensity)							
**HPV16 Status**	**N**	**Minimum**	**25th Pctl**	**Median**	**75th Pctl**	**Maximum**	***p*-value**
Negative	83	0	1	1.33	2.33	3	0.0115
Positive	110	0	1.33	2	2.33	3	
**p16 Status**							
Negative	47	0	1	1	2	3	0.0036
Positive	146	0	1	2	2.67	3	

DEK (Stain Proportion)							
**HPV16 Status**	**N**	**Minimum**	**25th Pctl**	**Median**	**75th Pctl**	**Maximum**	***p*-value**
Negative	83	0	30	75	90	100	0.0087
Positive	110	0	60	84.17	98.33	100	
**p16 Status**							
Negative	47	0	23.33	53.33	80	100	<.0001
Positive	146	0	60	85.83	100	100	

DEK (Quick Score)							
**HPV16 Status**	**N**	**Minimum**	**25th Pctl**	**Median**	**75th Pctl**	**Maximum**	***p*-value**
Negative	83	0	40	100	200	300	0.0046
Positive	110	0	80	160	206.67	300	
**p16 Status**							
Negative	47	0	33.33	63.33	150	300	<.0001
Positive	146	0	80	153.33	225	300	

Supplementary Table 3: DEK expression is associated with an increased hazard of death in HPV16+ but not HPV16- disease (survival univariate models)

Predictor	Hazard Ratio	95% CI		*p*-value	*N*
**HPV16 Negative**					
DEK Stain Intensity	0.763	0.568	1.026	0.0738	83
DEK Stain Proportion	0.996	0.989	1.004	0.3372	83
DEK Quick Score	0.998	0.995	1.001	0.1234	83
**HPV16 Positive**					
DEK Stain Intensity	1.535	0.975	2.416	0.0642	110
DEK Stain Proportion	1.013	0.999	1.028	0.0765	110
DEK Quick Score	1.004	1.000	1.008	**0.0339**	110

Supplementary Table 4: Clinical characteristics associated with DEK staining, based on HPV/p16 status (DEK stain proportion). See Supplementary_Table_4

Supplementary Table 5: DEK is not correlated with survival in p16 mono-labeled tumors (survival univariate models)

Predictor	Hazard Ratio	95% CI		*p*-value	*N*
p16 Positive					
DEK Stain Intensity	1.134	0.821	1.567	0.4451	146
DEK Stain Proportion	1.007	0.997	1.018	0.1443	146
DEK Quick Score	1.002	0.999	1.005	0.2534	146
p16 Negative					
DEK Stain Intensity	0.666	0.429	1.034	0.0704	47
DEK Stain Proportion	0.993	0.982	1.005	0.2471	47
DEK Quick Score	0.997	0.993	1.002	0.2579	47
